# Unveiling the mechanisms of yanhusuo’s therapeutic effects in neuropathic pain through network pharmacology, single-cell RNA sequencing, and molecular docking

**DOI:** 10.1186/s41065-025-00551-z

**Published:** 2025-09-24

**Authors:** Rui Liu, Min Yu, Kaihan Zhuang, Tingting Liu, Shanlian Suo, Haitao Dong

**Affiliations:** 1 Department of Anesthesiology, The Second Hospital of Lanzhou University, Lanzhou, 730030 Gansu China; 2 Southern Medical University, Guangzhou, 510515 Guangdong China; 3 Department of Orthopedics, The Second Hospital of Lanzhou University, Lanzhou, 730030 Gansu China

**Keywords:** Neuropathic pain, Yanhusuo, Network pharmacology, Molecular docking, Single-cell RNA sequencing, Multitarget therapy

## Abstract

**Background:**

Current therapeutic strategies for neuropathic pain (NP) encompass pharmacological agents, physical modalities, psychological support, and interventional procedures, which aim to mitigate inflammation, enhance vascular perfusion in afflicted regions, and modulate immune responses. However, the heterogeneity of NP pathogenesis and individual variability often lead to inconsistent treatment outcomes.

**Methods:**

An integrative network pharmacology framework was employed to elucidate the mechanistic basis of Yanhusuo in NP management. NP patients were categorized via unsupervised clustering, followed by single-cell sequencing and cell-cell communication analysis to identify immune cell interactions. Active compounds and targets of Yanhusuo were identified using the Traditional Chinese Medicine Systems Pharmacology (TCMSP) and SwissTargetPrediction databases. Network pharmacology tools, including Cytoscape, facilitated the construction of protein-protein interaction (PPI), compound-target-disease, and compound-target-pathway networks. Topological analyses identified core targets and pathways, while the Database for Annotation, Visualization and Integrated Discovery (DAVID) bioinformatics platform was used for functional enrichment analysis. Finally, molecular docking analysis was conducted to evaluate ligand-receptor binding affinities.

**Results:**

Nine bioactive compounds and 53 NP-associated targets were identified in Yanhusuo. PPI analysis suggests that ACTB, PPP1CA, ERK1, and PTEN may be the hub nodes with maximal centrality. KEGG pathway enrichment highlighted the focal adhesion pathway as pivotal in Yanhusuo’s anti-NP activity. Molecular docking suggests that there may be strong binding interactions between key compounds and hub targets (e.g. binding energy<-6.5 kcal/mol).

**Conclusions:**

This work systematically maps Yanhusuo’s multi-target, multi-pathway therapeutic landscape in NP, offering a strategic foundation for mechanistic research and drug discovery. The identified bioactive candidates represent promising candidates for NP therapeutics.

**Supplementary Information:**

The online version contains supplementary material available at 10.1186/s41065-025-00551-z.

## Background

Neuropathic pain (NP) is a chronic and often refractory condition arising from damage or dysfunction of the somatosensory nervous system, associated with spontaneous pain episodes, heightened nociceptive responses, and sensory abnormalities [1]. This complex neurological disorder manifests as both a protective physiological response and a maladaptive pathological state following neural injury [2]. Current first-line pharmacological treatments for neuropathic pain (e.g., gabapentinoids, antidepressants, opioids) are often limited by suboptimal efficacy in a substantial proportion of patients, significant adverse effects (e.g., dizziness, somnolence, dependency risk), and/or poor long-term tolerability. This leaves a significant unmet clinical need for safer and more effective therapeutic options [3]. This therapeutic gap mirrors historical challenges in oncology, where single-target therapies often succumb to compensatory pathway activation. As emphasized by Sonkin et al., the field’s progression toward *multi-target combinations* (e.g., kinase inhibitor cocktails in cancer) and *pathway-centric pharmacology* (e.g., PI3K-Akt axis modulation) has redefined therapeutic design for complex diseases [4].

The sensitization of primary sensory neurons in the dorsal root ganglion (DRG) is widely thought to cause the occurrence and persistence of hyperalgesia [5]. Recent research demonstrates that pain sensitivity emerges from the complex interplay of infiltrating immune cells and the nervous system. These immune cells, particularly macrophages, neutrophils, and lymphocytes, secrete various mediators that modulate both peripheral nociceptors and central neural circuits [6, 7]. The inflammatory factors produced serve as crucial regulators of nociceptive signaling and pain sensitization processes [8, 9]. Macrophages, which function as essential non-neuronal immune cells, maintain physiological equilibrium across diverse tissue environments [10, 11]. Despite extensive research on macrophages in NP, the functional subtypes and their interactions with other cells (especially neurons) remain unclear.

The advent of single-cell RNA sequencing (scRNA-seq) and its analytical methodologies has revolutionized the characterization of immune cell heterogeneity within the microenvironment, offering unprecedented insights into molecular dynamics [12]. Concurrently, network pharmacology, an interdisciplinary paradigm integrating bioinformatics, pharmacology, and systems biology, enables a more comprehensive exploration of traditional Chinese medicine (TCM) by mapping interactions between natural compounds and disease targets [13]. TCM, composed of numerous bioactive constituents, inherently function through multi-target mechanisms. This characteristic positions them as compelling candidates for addressing the polygenic and multi-pathway nature of NP potentially offering synergistic therapeutic effects while mitigating the risks associated with highly selective single-target drugs [14, 15], exemplified by Yanhusuo (*Corydalis* yanhusuo W.T. Wang), whose dried tuber has been used for centuries in powdered or decoction form. Yanhusuo is renowned for its analgesic and anti-inflammatory properties, attributed primarily to bioactive alkaloids [16]. Preclinical studies have highlighted its broad pharmacological effects, including modulation of nervous, digestive, and cardiovascular systems, as well as efficacy against acute, inflammatory, and NP without inducing tolerance [16, 17]. Despite these advances, the precise mechanisms underlying Yanhusuo’s therapeutic actions in NP remain incompletely defined, necessitating integrative approaches to unravel its molecular interplay with pain pathways.

**Fig. 1:**
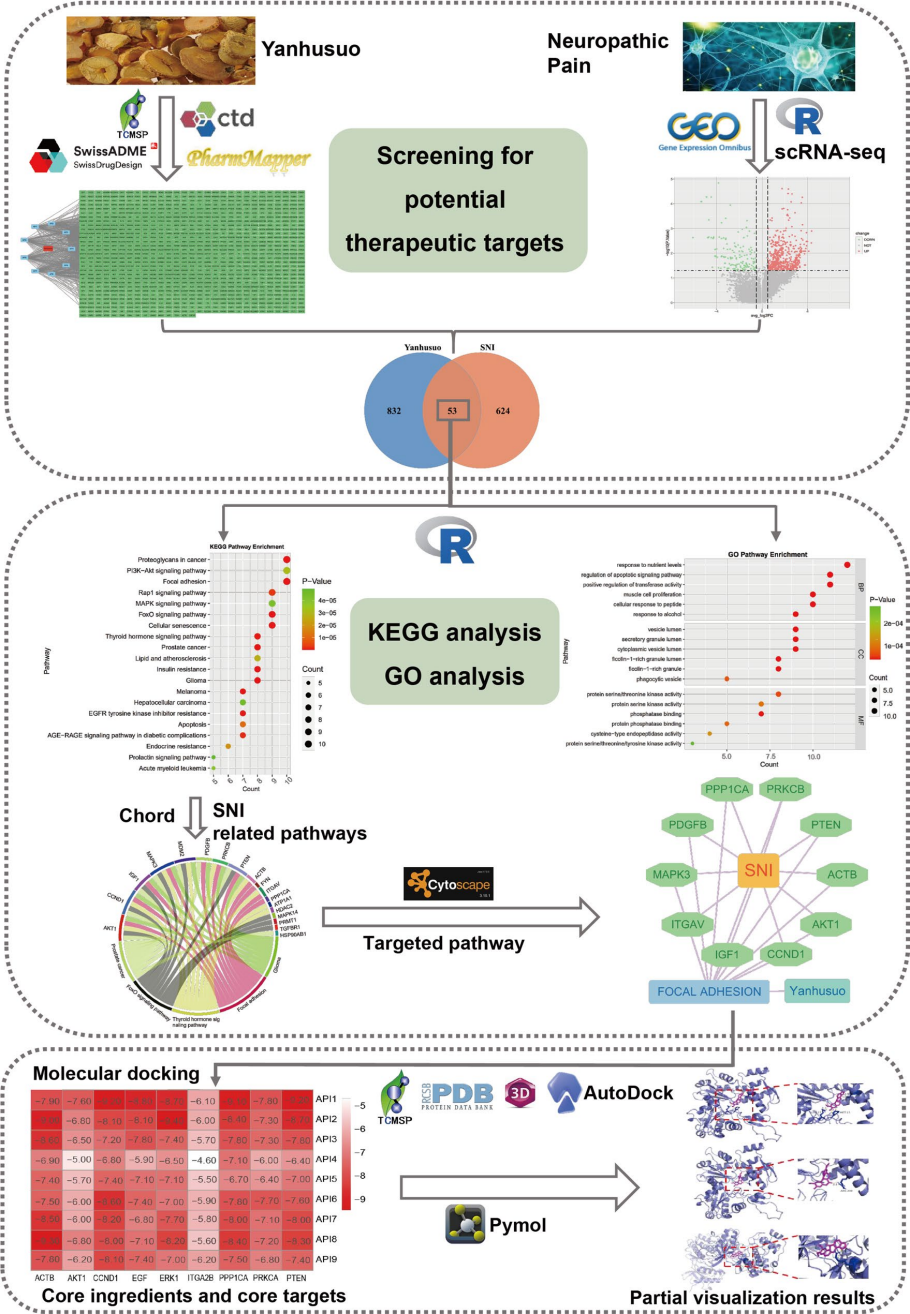
The workflow of the present study

In the present study, scRNA-seq analysis of spared nerve injury (SNI) models was conducted to elucidate macrophage-specific molecular signatures and their corresponding marker genes. We integrated this approach with network pharmacology and molecular docking analyses to systematically investigate the therapeutic targets and underlying mechanisms of Yanhusuo in NP treatment. This comprehensive methodology offers valuable insights for both drug development and clinical applications of Yanhusuo in NP management. The workflow of this study is presented in Fig. 1.

**Fig. 2:**
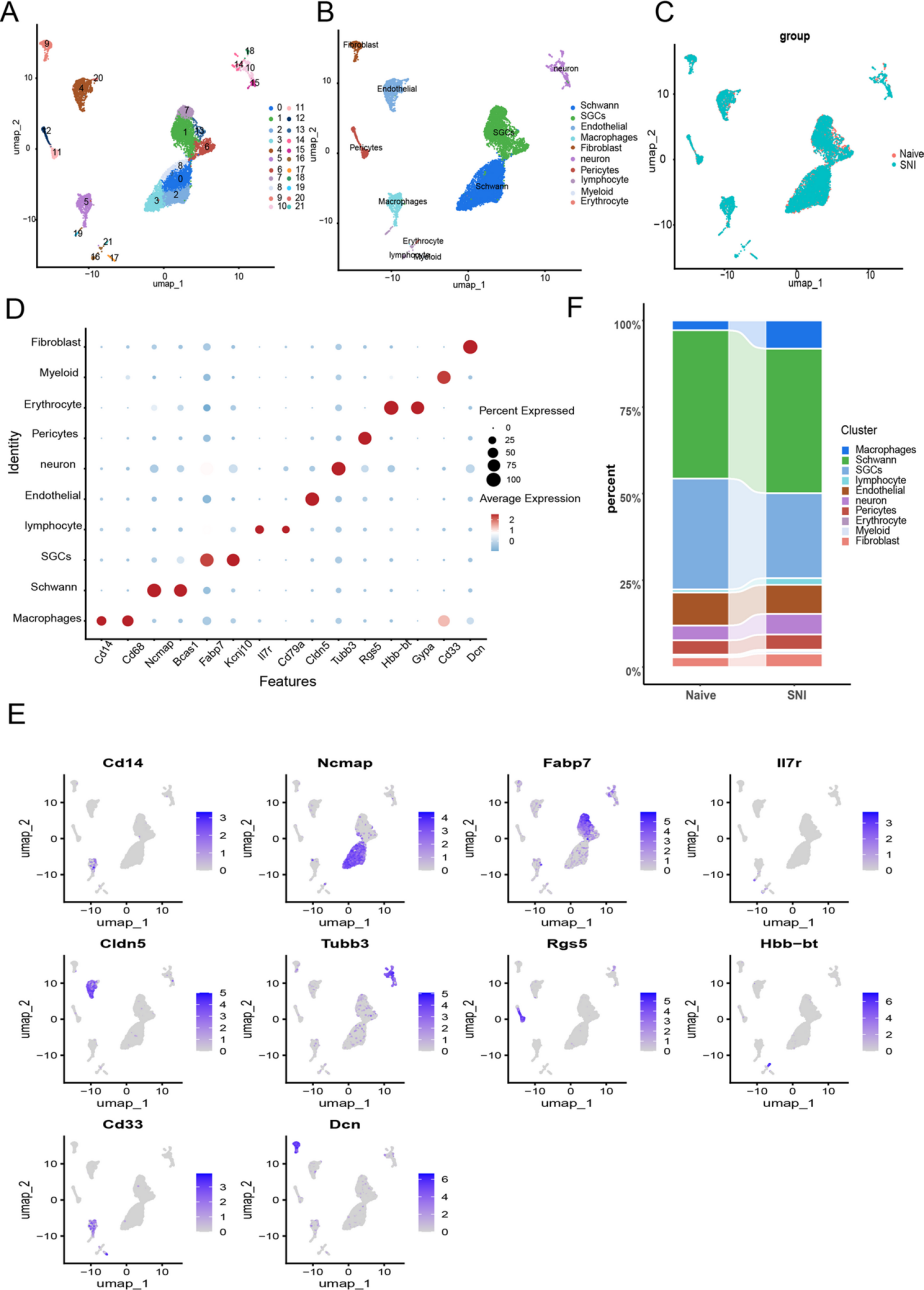
Single-cell atlas of SNI and naive DRG samples. **A**. UMAP plot colored by various cell clusters. **B**. UMAP plot colored by the subpopulation of cells after annotation. **C**. UMAP plots of the SNI group and naive group. **D**. Dot plot of selected marker genes for each cell type. The dot size represents the percentage of cells expressing each gene, while the dot color represents the level of expression. **E**. Feature plots of selected marker genes for each cell type. The color legend shows the log1p normalized expression levels of the genes. **F**. Bar plot depicting the proportions of the ten main cell types in the SNI and naive groups

**Fig. 3:**
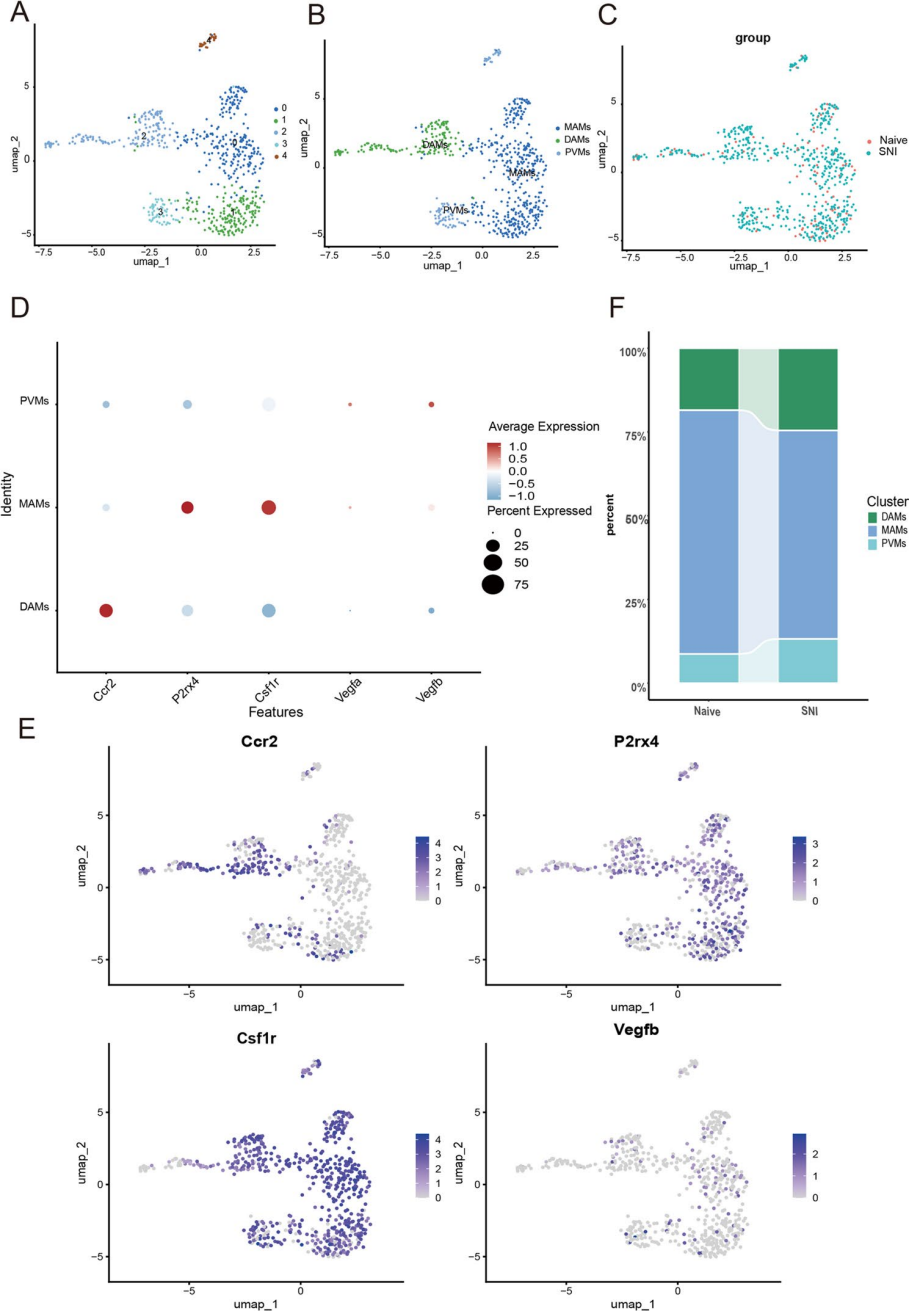
Alterations in Macrophage cell subtypes during DRG. **A**. UMAP plot colored by various macrophage cell clusters. **B**. UMAP plot colored by the subpopulation of cells after annotation. **C**. UMAP plots of the SNI group and naive group. **D**. Dot plot of selected marker genes for each macrophage cell type. The dot size represents the percentage of cells expressing each gene, while the dot color represents the level of expression. **E**. Feature plots of selected marker genes for each macrophage cell type. The color legend shows the log1p normalized expression levels of the genes. **F**. Bar plot depicting the proportions of the three main macrophage cell types in the SNI and naive groups

**Fig. 4:**
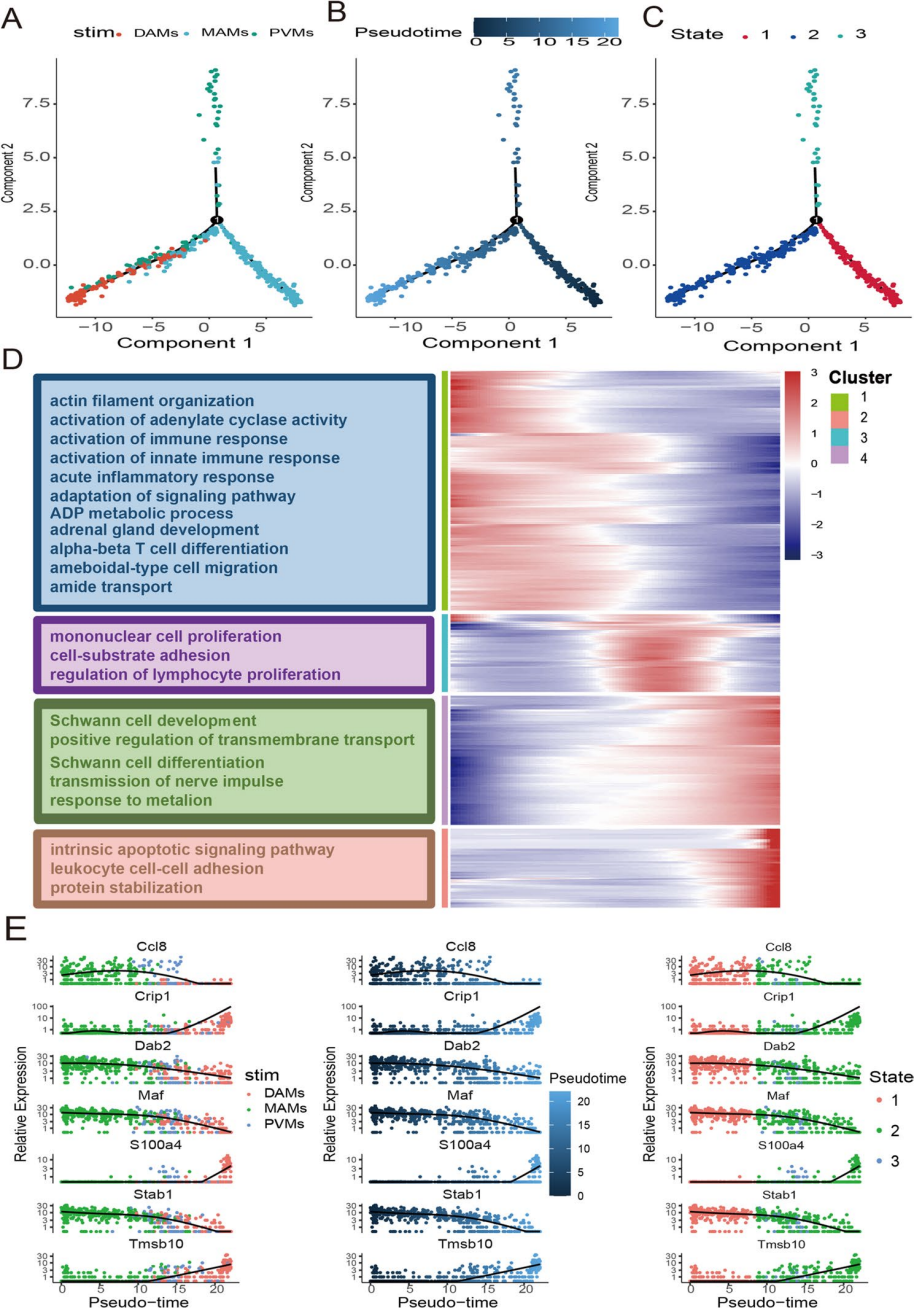
Simulation of the development trajectory of macrophage and the analysis of gene expression pattern inferred by Monocle2. **A**. The Monocle pseudotime trajectory plot shows the progression of three macrophage subclusters. **B**. Monocle pseudotime trajectory plot of macrophages presenting the beginning and end pseudotime profiles. **C**. The macrophage subcluster trajectory was separated into three cell states. **D**. The DEGs (in rows, q-value < 10 − 10) along the pseudotime were hierarchically clustered into four subclusters. The top annotated GO terms in each cluster were provided. **E**. Pseudotime kinetics of the top three genes among the three macrophage subclusters. Each dot represents a cell, different colors represent different clusters, and the ordinate represents the expression level of each gene

**Fig. 5:**
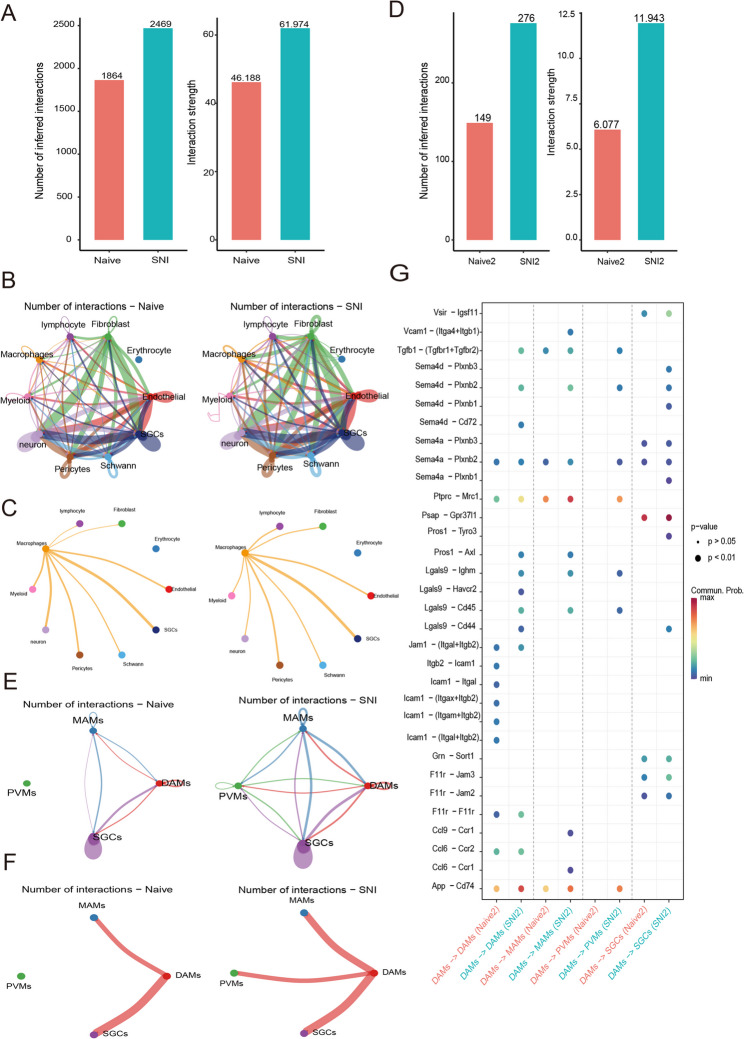
Communication network difference between the naive and SNI tissue. **A**. Comparison of the number and intensity of cell communications between SNI and naive tissues. **B**. Network interaction profile comparing naive and SNI tissues. Edge thickness corresponds to interaction frequency between cell populations. **C**. Quantitative representation of macrophage interaction networks between naive and SNI tissue. Edge thickness corresponds to interaction frequency between cell populations. **D**. Comparison of the quantity and intensity of subtypes of macrophage cell communication between SNI and naive tissues. **E**. Quantitative representation of macrophage interaction networks between subtypes of macrophages and SGCs in Naive and SNI tissues. Edge thickness corresponds to interaction frequency between cell populations. **F**. Quantitative representation of macrophage interaction networks between DAMs and SGCs in Naive and SNI tissues. Edge thickness corresponds to interaction frequency between cell populations. **G**. Key ligand-receptor pairs with significant changes in naive and SNI tissues

**Fig. 6:**
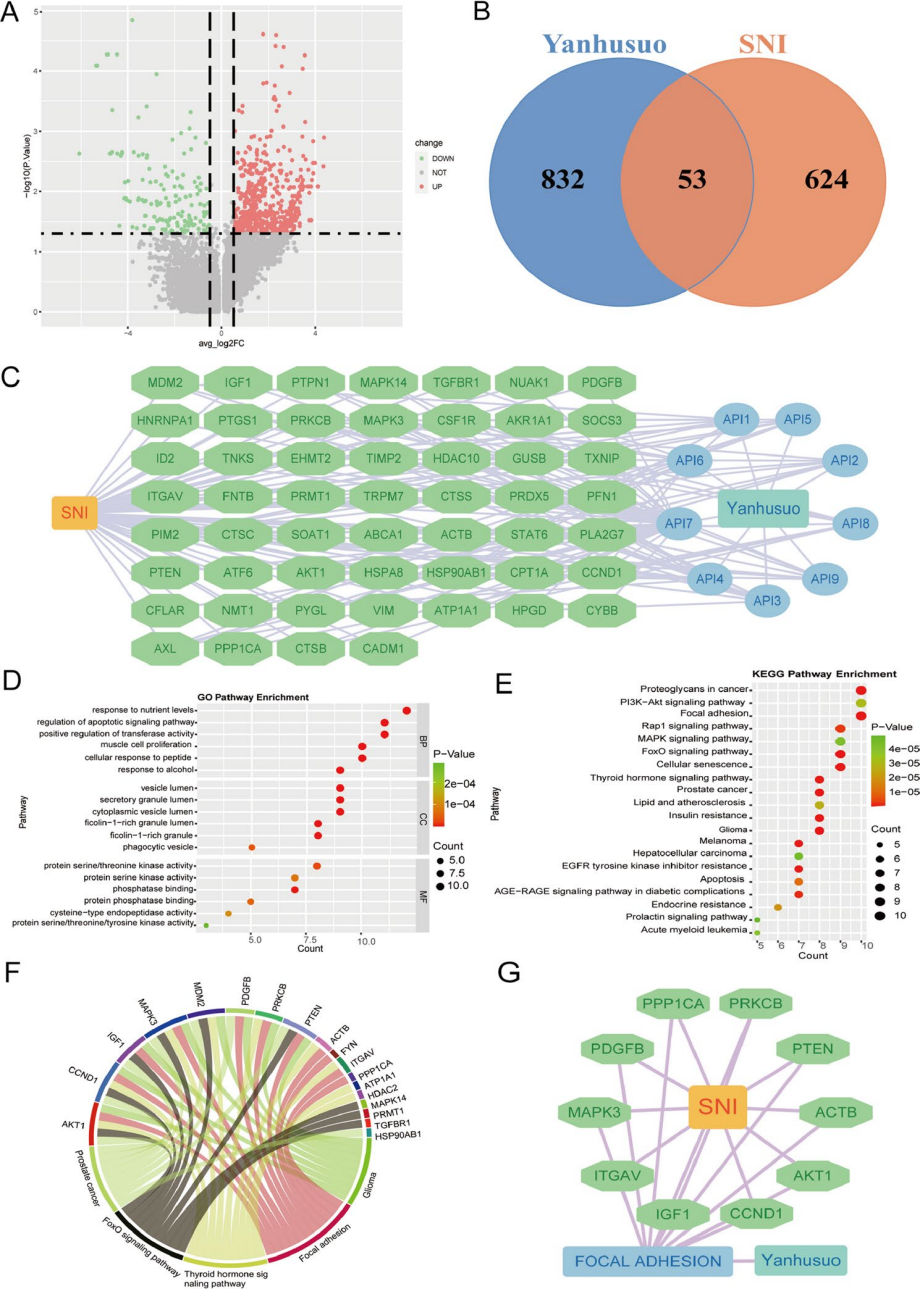
Identification of Macrophage-Related and Yanhusuo Targets in NP and Comprehensive Anti-NP Pathway Analysis. **A**. Volcano plot of differential analysis between macrophage groups, highlighting significant differences in gene expression between groups. **B**. Venn plot visualizing the common targets between drug targets and SNI cell-related targets. **C**. The “Drug-Active Component-Intersecting Gene-Disease” network. The blue green rectangle represents Yanhusuo, the light blue ellipses indicate active components, the green ellipses denote intersecting genes, and the orange rectangles represent SNI. **D**. GO enrichment analysis results, displaying the top 5 representative biological processes (BPs), cellular components (CCs), and molecular functions (MFs) with the lowest *p*-values (*P* < 0.05). **E**. KEGG pathway enrichment analysis identified 20 significant pathways associated with intersected genes (*P* < 0.05). **F**. Chord plot of the five key pathways involved in signal transduction. **G**. The “Drug-Pathway-Common Gene-Disease” network. The blue green rectangle represents Yanhusuo, the blue rectangle denotes pathways, the green ellipses indicate common genes, and the orange rectangles represent SNI

**Fig. 7:**
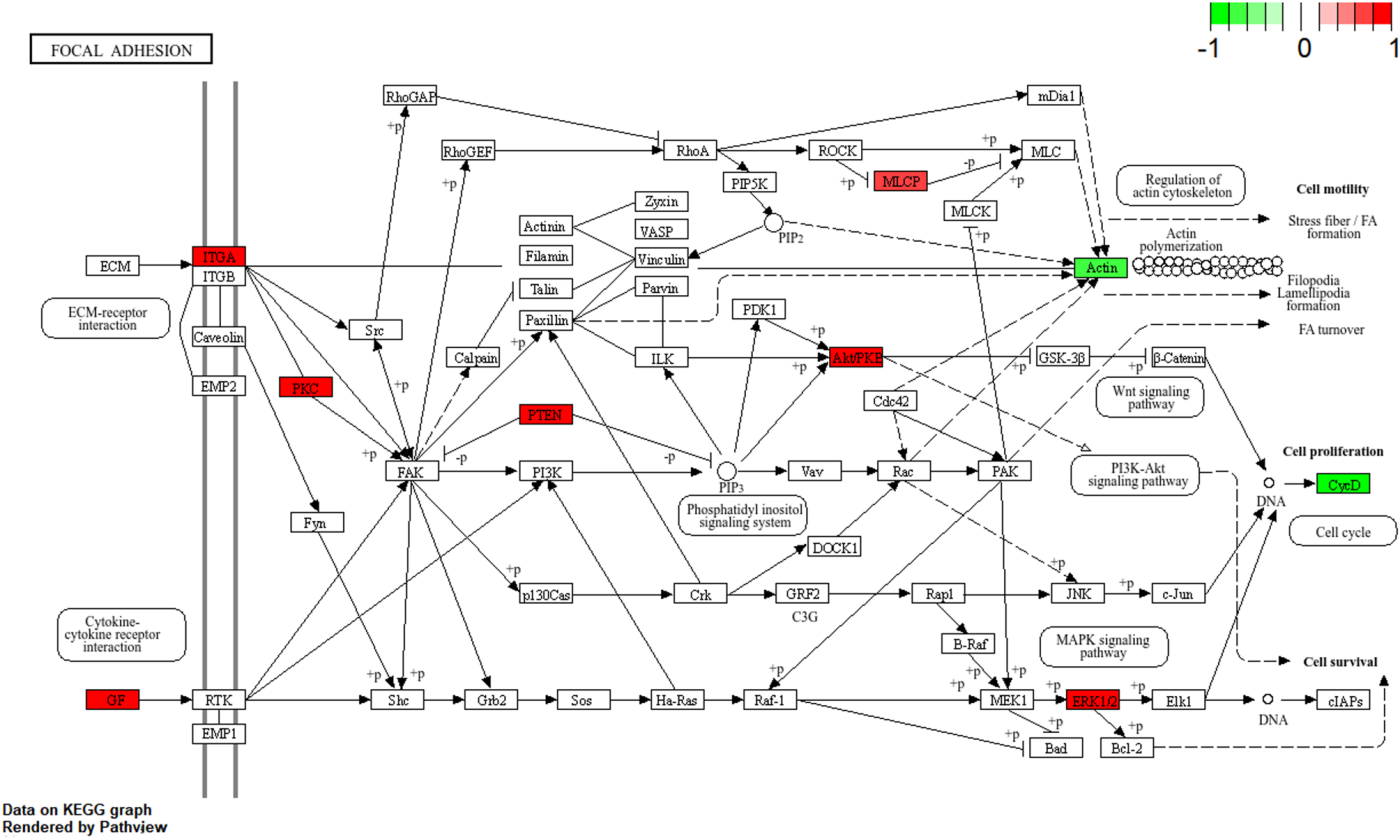
Focal adhesion signaling pathway. The core targets of Yanhusuo and SNI are marked in red and green. Red represents upregulated genes, green represents downregulated genes

**Fig. 8:**
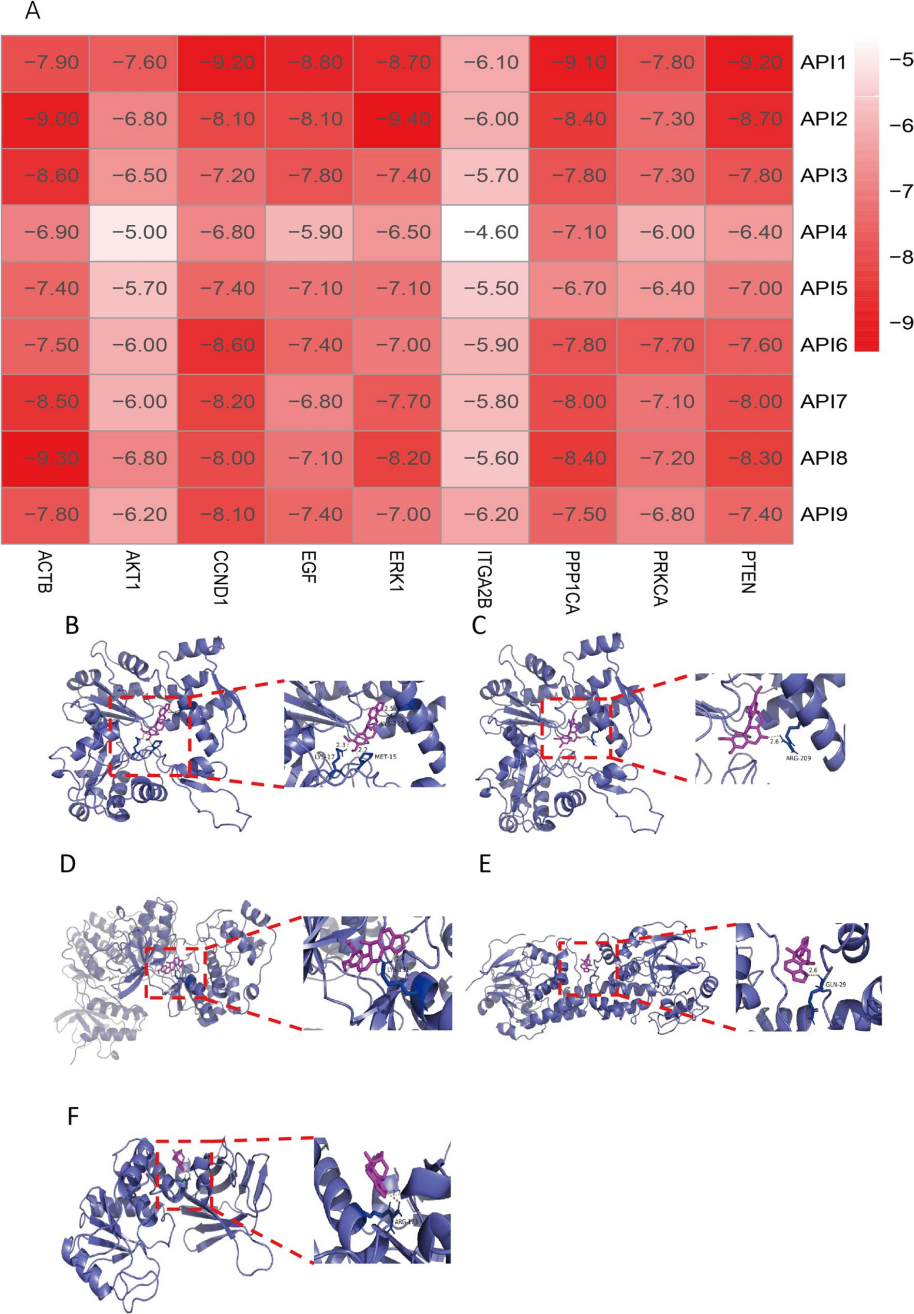
Molecular docking results for Yanhusuo’s active components and NP. **A**. Heat map of molecular docking results. **B**. Molecular docking results of dehydrocavidine and ACTB (affinity: -9.00). **C**. Molecular docking results of saulatine and ACTB (affinity: -9.30). **D**. Molecular docking results of dehydrocavidine andERK1 (affinity: -9.40). **E**. Molecular docking results of corynoloxine and PPP1CA (affinity: -9.10). **F**. Molecular docking results of corynoloxine and PTEN (affinity: -9.20)

## Methods

### Data acquisition

Single-cell RNA sequencing (scRNA-seq) data for dataset GSE174430 were retrieved from the publicly accessible Gene Expression Omnibus (GEO) repository (https://www.ncbi.nlm.nih.gov/geo/). In this study, 12 pairs of SNI and naive tissue samples from 6 mice were selected as raw data. As previously reported by Jager et al. [18], the dataset comprises gene-barcode matrices, feature annotations, and UMI-based barcode count tables. Ethical review was waived for this study since all analyzed data were anonymized and openly available.

### Dimension–reduction and cell clustering

During quality control, cells exhibiting mitochondrial gene content > 25% or UMI counts outside the 700–15,000 range were excluded. From the normalized expression matrix, the 2000 most highly variable genes (HVGs) were selected for principal component analysis (PCA). Jackstraw analysis and heatmap visualization of principal components (PCs 1–40) identified statistically significant PCs. Graph-based clustering (resolution = 0.5) was performed using the first 15 PCs to delineate cell subpopulations. Dimension reduction coordinates were computed via Seurat’s FindNeighbors and FindClusters functions, followed by Uniform Manifold Approximation and Projection (UMAP) for cluster visualization. Potential doublets and rare higher-order multiplets were filtered using DoubletFinder (v2.0.3), while batch effects were mitigated via Harmony (v1.0).

### Cell annotation

As previously reported, primary cell clusters were annotated through manual curation of canonical marker genes and their known functions [18]. Ambiguous cell subpopulations were provisionally designated using numerical identifiers (e.g., Subcluster 1, Subcluster 2) rather than alphanumeric codes to facilitate preliminary analysis. The dot plot of cell-specific markers was generated using the Seurat DotPlot function.

### Differentially expression analysis

Differentially expressed genes (DEGs) across cell clusters were identified using Seurat’s FindMarkers function. Statistical significance of inter-group expression differences was assessed via the Wilcoxon rank-sum test, with Bonferroni adjustment to control family-wise error rates. To account for multiple hypothesis testing, Benjamini–Hochberg correction was applied, and the false discovery rate (FDR) was quantified using adjusted q-values. DEGs were defined by three stringent thresholds: (1) Benjamini–Hochberg-adjusted Wilcoxon q-value ≤0.01; (2) absolute log2-transformed fold change (log2 FC) ≥0.50, calculated as the ratio of mean expression levels between compared groups; and (3) gene expression in ≥10% of cells within the target cluster.

### Pseudotime trajectory analysis

Pseudotemporal trajectories were reconstructed using Monocle2 (v2.14.0) to elucidate transcriptional dynamics underlying cellular state transitions across primary and subclustered cell populations [2]. Dimensionality reduction was achieved via the DDRTree algorithm, while pseudotemporal ordering of cells was implemented through Monocle2’s integrated trajectory inference framework. To identify genes dynamically regulated along developmental trajectories, the top 500 cluster-specific markers were subjected to differential expression analysis using the *differentialGeneTest* function, with pseudotime incorporated as a covariate in the full model formula. Expression trends were visualized using *plot_pseudotime_heatmap*, and genes were partitioned into four coherent modules (*num_clusters* = 4) based on shared pseudotemporal expression profiles and statistical significance.

### Cell-cell interaction analysis with cellchat

Intercellular communication networks were inferred using the CellChat package [19], leveraging its curated ligand-receptor interaction database (CellChatDB) [19]. Transcriptional count data were processed in CellChat under default settings, with secreted signaling pathways prioritized for analysis. A priori network knowledge was derived from predefined human protein-protein interaction (PPI) databases. Core computational workflows included the “computeCommunProbPathway” and “computeCommunProb” functions to quantify pathway and ligand-receptor-based communication probabilities, followed by network aggregation via “aggregateNet”. The statistical significance of inter-group interaction differences was assessed using a fixed randomization approach, with significance thresholds set at *p* < 0.05.

### Screening of active compounds of Yanhusuo

Bioactive components of Yanhusuo were systematically identified through the Traditional Chinese Medicine Systems Pharmacology Database (TCMSP; https://tcmsp-e.com/), a comprehensive repository integrating herb-compound-target-disease associations and pharmacokinetic profiles [20]. TCMSP catalogs physicochemical properties critical for drug-likeness evaluation, including oral bioavailability (OB), blood-brain barrier permeability, and molecular similarity to established therapeutics. Compounds were filtered using stringent thresholds: OB ≥ 30% (indicating systemic absorption efficiency) [19] and drug-likeness (DL) ≥ 0.18 (reflecting structural and functional congruence with approved drugs) [21, 22]. The DL metric evaluates molecular features, including functional group composition and physicochemical parameters against established drug databases [22], while OB quantifies the fraction of orally administered compounds reaching the systemic circulation [19]. Compounds satisfying both criteria were prioritized as putative bioactive compounds [23]. Corresponding molecular targets were predicted using TCMSP-linked gene annotations, supplemented by cross-referencing the Comparative Toxicogenomics Database (CTD; https://ctdbase.org/), SwissTargetPrediction (http://swisstargetprediction.ch/), and UniProt (https://www.uniprot.org/) to ensure comprehensive target mapping [24].

### Target identification of active ingredients

The intersection of targets between SNI-associated genes and Yanhusuo-predicted candidates was obtained using Venny 2.1.0 (http://bioinfo.cnb.csic.es/tools/venny/). Overlapping genes were subsequently analyzed using the STRING database (v11.5; http://string-db.org) [25]. with the search restricted to *Homo sapiens* and protein-protein interaction confidence thresholds set at ≥0.7. The resulting interaction network was imported into Cytoscape 3.7.1 (https://www.cytoscape.org/) for topological parameter calculation and identification of hub nodes through centrality metrics (e.g., degree, betweenness) [26].

### Functional enrichment analysis

Functional enrichment analysis of overlapping genes was performed via Gene Ontology (GO) and Kyoto Encyclopedia of Genes and Genomes (KEGG) pathways using the DAVID bioinformatics platform (v6.8; https://david.ncifcrf.gov) [27]. Statistical significance thresholds were set at a false discovery rate (FDR) < 0.05 and raw *p*-value < 0.05. Enrichment results were visualized using the R statistical environment (v3.4.1; https://www.r-project.org/), with customized ggplot2 scripts to generate publication-quality bar plots and bubble charts.

### Network construction

A herb-compound-target interaction network was constructed using Cytoscape 3.7.1 to systematically map the interactions between bioactive constituents and their molecular targets. Besides, to map multi-scale interactions across molecular, cellular, and systemic levels, a compound-target-pathway triad network was generated and analyzed in Cytoscape 3.7.1, integrating pathway enrichment data with ligand-receptor associations.

### Molecular docking

We next performed molecular docking simulations to evaluate interactions between nine key bioactive compounds from Yanhusuo and nine selected target proteins using AutoDockTools 1.5.6 and AutoDock Vina 4.2 [28] as follows: (1) Compound Preparation: Structural files (mol2 format) of core compounds were retrieved from the TCMSP database. These files were converted to 3D conformations in ChemOffice 20.0, followed by energy minimization, hydrogenation, and pdbqt format conversion via AutoDockTools 1.5.6. (2) Protein Structure Processing: Crystal structures of target proteins were obtained from the RCSB Protein Data Bank (https://www.rcsb.org/) [29]. Structures were imported into PyMOL 1.7.2.1 (https://pymol.org/2/) for dehydration, hydrogenation, and co-crystallized ligand removal. Docking grid boxes were centered on active sites using AutoDockTools and exported in pdbqt format. (3) Ligand-Receptor Docking: AutoDock Vina 4.2 executed the molecular docking simulations, calculating binding free energies between compounds and targets. (4) Interaction Analysis: PyMOL 2.5.2 was employed to visualize binding poses and quantify intermolecular interactions (e.g., hydrogen bonds, hydrophobic contacts) [30].

## Results

### NP single-cell transcriptome atlas

Single-cell RNA sequencing analysis was performed on 12 samples from dataset GSE174430, comprising SNI tissues from six mice and matched naive controls. After rigorous quality control filtering (retaining 9,867 high-quality cells), UMAP dimensionality reduction yielded 22 distinct cell clusters (Fig. 2A). Leveraging canonical marker gene expression (Fig. 2D) and prior annotation frameworks, we classified DRG immune cells into ten major populations (Fig. 2B): Macrophages (*Cd14+*,* Cd68+*), Schwann cells (*Ncmap+*), Satellite glial cells (SGCs) (*Fabp7+*), Lymphocytes (*II7r+*), Endothelial cells (*Cldn5+*), Neurons (*Tubb3+*), Pericytes (*Rgs5+*), Erythrocytes (*Hbb-bt+*), Myeloid cells (*Cd33+*), and Fibroblasts (*Dcn+*) (Fig. 2D, E ). Stratification of samples into SNI and naive groups revealed consistent UMAP clustering patterns between cohorts (Fig. 2C). Quantitative analysis demonstrated an approximate 3.5-fold enrichment in the proportion of macrophages in SNI tissues compared to naive controls (Fig. 2F).

### Single-cell transcriptome profiling of macrophage cells using scRNA-seq

Macrophage heterogeneity in the DRG exhibited pronounced disease-associated remodeling. Following dimensionality reduction analysis, 630 DRG macrophages were partitioned into five transcriptionally distinct subclusters (Fig. 3A). Subtype annotation (Fig. 3B) identified three functionally characterized populations: myelin-associated macrophages (MAMs; P2rx4+, Csf1r+) [31–33], disease-associated microglia (DAMs; Ccr2+) [34, 35], and perivascular macrophages (PVMs; Vegfb+) [36, 37]. UMAP projections confirmed consistent clustering patterns between the SNI and naive cohorts (Fig. 3C), with subtype-specific marker expression validated across groups (Fig. 3D, E). Notably, the SNI group exhibited an approximate 0.75-fold increase in the abundance of DAMs compared to the Naive group (Fig. 3F), underscoring their role in neuropathic pain pathogenesis.

### Pseudotime trajectory

To delineate the functional dynamics of macrophage subpopulations in NP, pseudotemporal trajectory analysis was employed. Monocle2-based reconstruction revealed three distinct macrophage states (Fig. 4A, B), with State 1 designated as the developmental origin (Fig. 4C). Genes exhibiting significant differential expression along the pseudotime axis were grouped into four distinct modules based on their expression trends, which are displayed in a heatmap (Fig. 4D): Module 1 (Green) comprised transiently upregulated genes with peak expression early in pseudotime, followed by a decline (*Dab2*,* Maf*,* Stab1*). Module 2 (Blue) encompassed genes with biphasic expression, with low initial expression, peaking mid-trajectory, then decreasing (*Ccl8*). Module 3 (Purple) consisted of late-activated genes showing progressive upregulation (*Crip1*,* Tmsb10*). Module 4 (Pink) included genes exhibiting sustained upregulation across pseudotime (*S100a4*). Pseudotemporal expression kinetics of seven key genes (*Ccl8*,* Crip1*,* Dab2*,* Maf*,* S100a4*,* Stab1*,* Tmsb10*) exhibited consistency with their module assignments (Fig. 4E), validating the temporal resolution of macrophage state transitions in NP.

### Cell communication analysis

Cell-cell communication networks in SNI tissues exhibited markedly elevated interaction frequencies and signal intensities relative to naive controls, as evidenced by differential network weights and connectivity metrics (Fig. 5A, D). This heightened intercellular crosstalk likely reflects amplified interplay between macrophage and SGCs and adaptive immune remodeling within the SNI microenvironment. Quantitative assessment of ligand-receptor probabilities across cell subpopulations revealed marked disparities between SNI and naive groups (Fig. 5B, C), with macrophage-SGC interactions emerging as a dominant axis of dysregulation (Fig. 5E, F). Systemic pathway interrogation (Fig. 5G) identified significant activation of six signaling axes in SNI: Lgals9-CD44 (galectin-9–CD44 axis), Pros1-Tyro3 (protein S–TAM receptor), Sema4a-Plxnb1 (semaphorin 4 A–plexin B1), Sema4d-Plxnb2/3 (semaphorin 4D–plexin B2/B3). These signaling pathways collectively orchestrate neuroimmune crosstalk, as evidenced by their enriched ligand-receptor pairings in SNI (Fig. 5G). Such dysregulated molecular interfaces constitute prospective targets for precision modulation of pathological cell communication in neuropathic pain.

### Genes and pathways involved in the interaction between drugs and diseases

Transcriptomic data from dataset GSE174430 were obtained from the GEO database, identifying 677 DEGs in SNI tissues-542 upregulated and 135 downregulated (Fig. 6A). The intersection of putative Yanhusuo-associated targets (n = 832) compiled from PharmMapper, CTD, and SwissTargetPrediction databases yielded 53 DEGs (Fig. 6B, C). Functional enrichment analysis of the 53 DEGs using the clusterProfiler R package revealed significant enrichment in six biological processes (BP) and KEGG pathways (Fig. 6D, E), including ‘Proteoglycans in cancer’, ‘PI3K − Akt signaling pathway’ and ‘Focal adhesion’, highlighting their pivotal roles in Yanhusuo’s therapeutic action. A multi-target network integrating these pathways was constructed in Cytoscape 3.8.2 (Fig. 6G), while chord plots (Fig. 6F) visualized interactions among five core pathways: Focal adhesion (mechanotransduction and neuronal survival), FoxO signaling (oxidative stress regulation), Thyroid hormone signaling (metabolic modulation), Prostate cancer (cell proliferation control), Glioma signaling (glia-neuron crosstalk). These pathways collectively delineate the central pharmacological mechanisms underlying Yanhusuo’s anti-NP effects.

### Focal adhesion signal pathway network diagram

The target pathway network was subsequently established based on pathways associated with Focal adhesion (Fig. 7), where key genes such as *ACTB*, *AKT1*, *CCND1*, *EGF*, *ITGA2B*, *PPP1CA*, *ERK1*, *PRKCA*, and *PTE* exhibited prominent topological ranking. The convergence of these pathways on shared targets suggests that Yanhusuo’s therapeutic effect on NP arises from multi-pathway regulation. An integrated pathway schematic was generated to illustrate the intricate network of Yanhusuo’s interactions involved in NP treatment. Within the context of NP, Yanhusuo primarily engages two signaling mechanisms: the Focal adhesion pathway and the PI3K − Akt signaling pathway. Fig. 7 highlights critical nodal points in these pathways, with components such as GF, ITGA, PKC, PTEN, MLCP, Akt/PKB, ERK1/2, CycD, and Actin demonstrating significant functional involvement.

### Molecular docking

Building upon prior findings, nine prioritized compounds—Corynoloxine (API1), dehydrocavidine (API2), dehydrocorobine (API3), leonticine (API4), methyl-[2-(3,4,6,7-tetramethoxy-1-phenanthryl)ethyl]amine (API5), pontevedrine (API6), quercetin (API7), saulatine (API8), and ST057701 (API9)—were analyzed against key molecular targets: ACTB, AKT1, CCND1, EGF, ITGA2B, PPP1CA, ERK1, PRKCA, and PTEN (Fig. 8A7). Binding affinities were interpreted using established thresholds: values below − 4.25 kcal/mol suggest detectable ligand-target interactions, while scores < − 5.0 kcal/mol and < − 7.0 kcal/mol denote progressively stronger binding capacities, respectively [38]. Computational screening procedures identified notable ligand-receptor binding affinities in specific molecular pairings: ACTB-API2 (− 9.00 kcal/mol), ACTB-API8 (− 9.30), ERK1-API2 (− 9.40), PPP1CA-API1 (− 9.10), and PTEN-API1 (− 9.20), with results visualized in Fig.  8.

## Discussion

NP affects an estimated 1–5% of the global population annually [39]. Characterized by high recurrence rates, NP arises from heterogeneous etiological factors and multifaceted pathological mechanisms involving numerous signaling pathways, leading to suboptimal clinical outcomes. These challenges often culminate in profound physiological and psychological burdens for patients, significantly compromising their quality of life [40–42]. While the pathophysiological basis of NP remains incompletely elucidated, recent research has prioritized understanding inflammatory cascades and immune microenvironment dysregulation in NP progression [6]. In this study, we adopted a multi-modal strategy that integrates database mining, network pharmacology, computational docking, molecular dynamics simulations, and single-cell transcriptomics to systematically identify the bioactive constituents of Yanhusuo and their interaction targets. This comprehensive approach facilitated an in-depth exploration of the herb’s putative mechanisms against NP.

At present, scRNA-seq has become a cornerstone in exploring immune cell dynamics, particularly in resolving macrophage heterogeneity and their regulatory interplay within the immune microenvironment [2, 43–45]. Our DRG cell atlas in SNI mice extends the paradigm of single-cell integration analyses for complex disease mechanisms. As exemplified by Siweier Luo et al.‘s landmark study revealing immune heterogeneity across five autoimmune diseases, scRNA-seq enables identification of pathogenic cell states inaccessible to bulk sequencing [46]. Similar to their discovery of IFN-responsive monocyte subsets in lupus [46], our work uncovers injury-specific macrophage subpopulations (e.g., Lgals9⁺ macrophages) driving neuro-immune crosstalk. This reinforces scRNA-seq’s power to resolve conserved inflammatory mechanisms across neurological and autoimmune disorders.

Pseudotime trajectory analysis classified dynamically expressed macrophage genes into four temporally regulated clusters, functionally enriched in lymphocyte-mediated immunity, immune cell proliferation, cytoskeletal remodeling (actin filament organization), and antigen processing/presentation. Cell-cell communication analysis revealed robust macrophage interactions with neighboring cell types, predominantly mediated by Sema4d, Pros1, and Lgals9 signaling axes. This finding aligned with a study by Li et al. [47], where chronic constriction injury (CCI) augmented glial crosstalk in spinal cord tissues while impairing function-associated receptor-ligand interactions, implicating microglia and astrocytes in oligodendrocyte differentiation arrest at the Oligo-2 stage post-CCI. Consistently, Feng et al. [48] demonstrated macrophage-derived prosaposin signaling through GPR37L1 receptors on SGCs, suggesting a neuroprotective dialog mediated by scRNA-seq-informed CellChat modeling. The above findings overlap in their assertion of the distinct antigen-presenting capacity of tissue-resident macrophages, which orchestrate microenvironment-specific cellular interactions to maintain immunological homeostasis.

Building on these findings, we utilized a systems-based pharmacological approach to map macrophage-associated pathogenic pathways and identify potential therapeutic targets. This approach, rooted in systems biology, leverages comprehensive network-based analyses of biological systems to identify pivotal signaling nodes, thereby facilitating the design of multi-target therapeutic agents [13]. TCM formulations typically exert their effects via multi-target and multi-level mechanisms, aligning with the systemic and integrative principles of network pharmacology [14]. Empirical evidence further supports this synergy; for example, Yuanhu Zhitong Formula—a classic TCM prescription—has demonstrated immunomodulatory properties, including enhanced macrophage activity, pathogen clearance, and microcirculation, contributing to its analgesic efficacy [49, 50]. It is now understood that the TCM herb Yanhusuo derives its pharmacological activity primarily from alkaloids. Beyond analgesic effects, these bioactive compounds modulate immune cell functions, exerting anti-inflammatory and immunoregulatory roles that may underlie therapeutic efficacy in disease management [15, 16, 51]. To elucidate the molecular and pharmacological mechanisms of Yanhusuo in NP, we applied this systems biology-derived methodology.

Using network pharmacology, we identified 9 bioactive compounds from Yanhusuo and 832 potential drug targets from the TCMSP and TCMID databases, applying ADME criteria for screening. Subsequently, 624 disease-associated targets linked to SNI were retrieved from the GEO dataset. The intersection of results between SNI and Yanhusuo yielded 53 DEGs.

SGCs, localized in the DRG, act as critical intermediaries in pain signal transmission [12, 52]. Following SNI, SGCs within the DRG undergo rapid activation mediated by gap junction communication and inflammatory factor release, establishing neuronal-SGC functional units that amplify neuronal excitability and drive central sensitization [53]. Activated SGCs further recruit peripheral macrophages to infiltrate the DRG by secreting chemokines [52]. Macrophage-derived Sema4d binds to Plxnb1 receptors on SGCs, initiating downstream signaling cascades such as PKC, ERK1/2, and Akt/PKB pathways [54–59]. Current evidence suggests that these pathways may induce transcriptional changes in focal adhesion-associated genes (e.g., *GF*, *ITGA*, *PTEN*) [60–63]. Post-injury, ligand-mediated activation of the LIgals9-Cd44 axis facilitates interaction of the CD44 intracellular domain with the actin cytoskeleton, disrupting actin dynamics and impairing cytoskeletal remodeling, cell morphology, and migration [64–66]. Dysregulation of focal adhesion pathways enhances cell-cell adhesion, exacerbates inflammatory responses, and amplifies pain signal propagation [60, 62, 63].

These findings collectively suggest that Yanhusuo’s bioactive components modulate multiple signaling axes, including Sema4d-Plxnb1/2/3, Lgals9-Cd44, and focal adhesion pathways, through ligand-receptor interactions. This multi-pathway involvement may attenuate NP by reducing inflammatory responses, regulating cell migration and cytoskeletal remodeling, and promoting tissue repair—mechanisms that collectively improve clinical prognosis [54–66]. However, the precise molecular interplay underlying these effects requires further experimental validation.

Finally, to elucidate the molecular mechanisms underlying the efficacy of Yanhusuo against NP, we integrated network pharmacology predictions with molecular docking simulations targeting nine key proteins (ACTB, AKT1, CCND1, EGF, ITGA2B, PPP1CA, ERK1, PRKCA, and PTEN) and TCMSP-derived bioactive compounds (e.g., corynoloxine, dehydrocavidine, saulatine). Binding energy assessments revealed enhanced receptor-ligand affinity and conformational stability at lower energy values, with a threshold of <-5 kcal/mol widely recognized as indicative of robust interactions [13, 67, 68]. Notably, Yanhusuo compounds exhibited high binding occupancy and low energy scores (<-5 kcal/mol) for ACTB, PPP1CA, ERK1, and PTEN, corroborating PPI network predictions and identifying these proteins as putative therapeutic targets. Structural analyses further highlighted hydrogen bonding and hydrophobic interactions as critical drivers of target engagement, suggesting that Yanhusuo alleviates NP through multi-target interactions.

Our study does not assert therapeutic causality but identifies high-confidence target-pathway hypotheses for experimental validation. The convergence of DEG-derived pathways, network pharmacology, and docking scores provides a tripartite filter to reduce false positives.

Our integrated network pharmacology and molecular docking approach not only delineated the potential multi-target mechanisms underlying Yanhusuo’s efficacy against neuropathic pain but also computationally prioritized key bioactive alkaloids (e.g., corynoloxine, dehydrocavidine, saulatine) interacting with critical targets within the focal adhesion pathways (e.g., PTEN, ITGA, Akt/PKB). The application of molecular docking in this context is particularly valuable for drug screening, especially in complex herbal systems [13]. It serves to refine network pharmacology predictions by providing structural insights into compound-target binding, thereby enhancing confidence in the identified interactions and focusing downstream experimental efforts on the most promising candidates. This strategy mirrors successful applications in drug repurposing, such as the identification of BMS345541 for glioblastoma through integrated virtual screening [69], and is increasingly recognized as a powerful tool for deciphering the ‘active ingredients group’ within traditional medicines [67].

Building upon this computational foundation, the logical next steps involve rigorous experimental validation to translate these in silico insights into biological and therapeutic reality. In vitro, the direct binding of prioritized Yanhusuo alkaloids (e.g., dehydrocorybulbine) to key targets like PTEN should be confirmed using biophysical techniques (e.g., SPR, ITC). Subsequently, their functional effects on focal adhesion dynamics, neuronal excitability, and neuroinflammation should be assessed in primary sensory neurons (e.g., DRG explants) and glial cells under conditions mimicking NP. In vivo, the efficacy of these key alkaloids or optimized Yanhusuo fractions needs evaluation in established rodent neuropathic pain models (e.g., CCI, SNI) using quantitative behavioral measures (mechanical allodynia, thermal hyperalgesia). Crucially, exploring biomarkers to track pathway modulation in vivo is essential. Measuring changes in phosphorylation states of focal adhesion pathway components in DRG or spinal cord tissue, or potentially in accessible biosamples like PBMCs, could provide valuable pharmacodynamic readouts correlating with therapeutic response.

It should be mentioned that this study had some limitations. First, bioactive compounds as well as target data were retrieved from the literature and databases; therefore, the reliability and accuracy of the predictions were dependent on data quality. Second, this study used a data mining approach, and clinical trials and animal experiments are needed to confirm the findings. However, the method we use provides a new approach for exploring traditional Chinese medicine treatment.

Looking towards clinical translation, the multi-target nature of Yanhusuo presents intriguing possibilities. Beyond developing Yanhusuo-derived preparations as standalone therapies, investigating potential synergistic interactions with existing neuropathic pain drugs (e.g., pregabalin, duloxetine) is a promising avenue. Preclinical studies exploring such combinations could reveal regimens offering enhanced efficacy or reduced dosages (and thus side effects) of conventional drugs. Ultimately, advancing these findings requires focused research on the pharmacokinetics, bioavailability (potentially addressing challenges with alkaloid absorption), and comprehensive safety profiles of the active Yanhusuo components. This progression from computational prediction (in silico) to experimental validation (in vitro and in vivo) and finally to clinical exploration represents a critical pathway for realizing the potential of multi-target herbal approaches like Yanhusuo in addressing the unmet needs of NP patients.

## Conclusions

NP represents one of the most prevalent forms of chronic pain globally, characterized by high incidence and recurrence rates. Yanhusuo exhibits a broad spectrum of therapeutic properties, including anti-inflammatory, immunomodulatory, analgesic, sedative, and cardioprotective effects. Experimental studies have shown that Yanhusuo mitigates neuroinflammation in rodent models by suppressing microglial M1 polarization and attenuating pro-inflammatory mediator release. However, a comprehensive understanding of its molecular targets and associated biological processes in NP treatment remains elusive. To address this knowledge gap, we employed multiple analytical approaches. Using GEO dataset mining and scRNA-seq, we proposed detailed transcriptome profiles of NP, with emphasis on macrophage-mediated cellular trajectories and intercellular communication mechanisms. Network pharmacology analysis suggests a potential computational evidence for potential Yanhusuo-Mediated pathway regulation through systematic evaluation of traditional Chinese medicine databases. This investigation identified 9 bioactive compounds and 832 potential targets, with 53 exhibiting overlap between Yanhusuo and SNI.

Further bioinformatic analyses, including PPI networks, GO, and KEGG pathway enrichment, revealed multiple biological processes may affected by Yanhusuo. Notably, KEGG analysis predicted the involvement of focal adhesion and PI3K-Akt signaling pathways in SNI treatment. Molecular docking studies supported good binding affinities between active compounds and their predicted targets. Our findings collectively predict that Yanhusuo may modulate inflammatory and immune responses through regulation of pro-inflammatory cytokines and signaling molecules. Overall, this comprehensive analysis provides novel insights into mechanisms underlying Yanhusuo’s efficacy against NP and identifies promising plausible candidates for therapeutic development.

## Supplementary Information

Below is the link to the electronic supplementary material.

Supplementary Material 1

## Data Availability

The sequencing data used to support the findings of this study have been deposited in the GEO repository (GSE174430).

## Abbreviations

NP: Neuropathic pain
PPI: Protein-protein interaction
DRG: Dorsal root ganglion
scRNA-seq: Single-cell RNA sequencing
TCM: Traditional Chinese medicine
SNI: Spared nerve injury
GEO: Gene expression omnibus
HVGs: Highly variable genes
PCA: Principal component analysis
PCs: Principal components
DEGs: Differentially expressed genes
FDR: False discovery rate
TCMSP: Traditional Chinese medicine systems pharmacology database
OB: Oral bioavailability
DL: Drug-likeness
CTD: Comparative toxicogenomics database
GO: Gene ontology
KEGG: Kyoto encyclopedia of genes and genomes
UMAP: Uniform manifold approximation and projection
MAMs: Myelin-associated macrophages
DAMs: Disease-associated microglia
PVMs: Perivascular macrophages
SGCs: Satellite glial cells
PKC: Protein kinase C

## References

[1] Baron R, Binder A, Wasner G. Neuropathic pain: diagnosis, pathophysiological mechanisms, and treatment. Lancet Neurol. 2010;9(8):807–19. 20650402 10.1016/S1474-4422(10)70143-5

[2] Deng Y, Tang S, Cheng J, Zhang X, Jing D, Lin Z, Zhou J. Integrated analysis reveals Atf3 promotes neuropathic pain via orchestrating JunB mediated release of inflammatory cytokines in DRG macrophage. Life Sci. 2023;329:121939. 37451398 10.1016/j.lfs.2023.121939

[3] Calvo M, Davies AJ, Hébert HL, Weir GA, Chesler EJ, Finnerup NB, Levitt RC, Smith BH, Neely GG, Costigan M, Bennett DL. The genetics of neuropathic pain from model organisms to clinical application. Neuron. 2019;104(4):637–53. 31751545 10.1016/j.neuron.2019.09.018 PMC6868508

[4] Sonkin D, Thomas A, Teicher BA. Cancer treatments: past, present, and future. Cancer Genet. 2024;286–287:18–24. 38909530 10.1016/j.cancergen.2024.06.002 PMC11338712

[5] Chung JM, Chung K. Importance of hyperexcitability of DRG neurons in neuropathic pain. Pain Pract. 2002;2(2):87–97. 17147683 10.1046/j.1533-2500.2002.02011.x

[6] Calvo M, Dawes JM, Bennett DL. The role of the immune system in the generation of neuropathic pain. Lancet Neurol. 2012;11(7):629–42. 22710756 10.1016/S1474-4422(12)70134-5

[7] Machelska H. Dual peripheral actions of immune cells in neuropathic pain. Arch Immunol Ther Exp (Warsz). 2011;59(1):11–24. 21234811 10.1007/s00005-010-0106-x

[8] Grace PM, Hutchinson MR, Maier SF, Watkins LR. Pathological pain and the neuroimmune interface. Nat Rev Immunol. 2014;14(4):217–31. 24577438 10.1038/nri3621 PMC5525062

[9] Liu JA, Yu J, Cheung CW. Immune actions on the peripheral nervous system in pain. Int J Mol Sci 2021;22(3). 10.3390/ijms22031448 PMC7867183 33535595

[10] Wynn TA, Chawla A, Pollard JW. Macrophage biology in development, homeostasis and disease. Nature. 2013;496(7446):445–55. 23619691 10.1038/nature12034 PMC3725458

[11] Murray PJ, Wynn TA. Protective and pathogenic functions of macrophage subsets. Nat Rev Immunol. 2011;11(11):723–37. 21997792 10.1038/nri3073 PMC3422549

[12] Chen L, Li Y, Zhu L, Jin H, Kang X, Feng Z. Single-cell RNA sequencing in the context of neuropathic pain: progress, challenges, and prospects. Transl Res. 2023;251:96–103. 35902034 10.1016/j.trsl.2022.07.004

[13] Li J, Wang D, Hao X, Li Y, Gao H, Fan Y, Fang B, Guo Y. Exploring the high-quality ingredients and mechanisms of Da Chuanxiong formula in the treatment of neuropathic pain based on network pharmacology, molecular docking, and molecular dynamics simulation. Biomed Pharmacother. 2024;178:117195. 39068852 10.1016/j.biopha.2024.117195

[14] Li SH, Li L, Yang RN, Liang SD. Compounds of traditional Chinese medicine and neuropathic pain. Chin J Nat Med. 2020;18(1):28–35. 31955821 10.1016/S1875-5364(20)30002-9

[15] Chen L, Chen J, Ramesh T, Seshadri VD, Zhu L. Zinc oxide nanoparticles from corydalis Yanhusuo attenuated the mycoplasmal pneumonia in mice through inhibiting the MAPKs signaling pathway. Microb Pathog. 2020;147:104270. 32446872 10.1016/j.micpath.2020.104270

[16] Wang L, Zhang Y, Wang Z, Gong N, Kweon TD, Vo B, Wang C, Zhang X, Chung JY, Alachkar A, Liang X, Luo DZ, Civelli O. The antinociceptive properties of the corydalis Yanhusuo extract. PLoS ONE. 2016;11(9):e0162875. 27622550 10.1371/journal.pone.0162875 PMC5021270

[17] Wu L, Zhang W, Qiu X, Wang C, Liu Y, Wang Z, Yu Y, Ye RD, Zhang Y. Identification of alkaloids from corydalis Yanhusuo W. T. Wang as dopamine D_1_ receptor antagonists by using CRE-Luciferase reporter gene assay. Molecules 2018;23(10). 10.3390/molecules23102585 PMC6222624 30308941

[18] Jager SE, Pallesen LT, Lin L, Izzi F, Pinheiro AM, Villa-Hernandez S, Cesare P, Vaegter CB, Denk F. Comparative transcriptional analysis of satellite glial cell injury response. Wellcome Open Res. 2022;7:156. 35950162 10.12688/wellcomeopenres.17885.1 PMC9329822

[19] Jin S, Guerrero-Juarez CF, Zhang L, Chang I, Ramos R, Kuan CH, Myung P, Plikus MV, Nie Q. Inference and analysis of cell-cell communication using cellchat. Nat Commun. 2021;12(1):1088. 33597522 10.1038/s41467-021-21246-9 PMC7889871

[20] Zheng M, Yang X, Yuan P, Wang F, Guo X, Li L, Wang J, Miao S, Shi X, Ma S. Investigating the mechanism of Sinisan formula in depression treatment: a comprehensive analysis using GEO datasets, network pharmacology, and molecular Docking. J Biomol Struct Dyn. 2025;43(5):2397–411. 38174416 10.1080/07391102.2023.2297816

[21] Xu X, Zhang W, Huang C, Li Y, Yu H, Wang Y, Duan J, Ling Y. A novel chemometric method for the prediction of human oral bioavailability. Int J Mol Sci. 2012;13(6):6964–82. 22837674 10.3390/ijms13066964 PMC3397506

[22] Tao W, Xu X, Wang X, Li B, Wang Y, Li Y, Yang L. Network pharmacology-based prediction of the active ingredients and potential targets of Chinese herbal radix curcumae formula for application to cardiovascular disease. J Ethnopharmacol. 2013;145(1):1–10. 23142198 10.1016/j.jep.2012.09.051

[23] Ban C, Jo M, Park YH, Kim JH, Han JY, Lee KW, Kweon DH, Choi YJ. Enhancing the oral bioavailability of Curcumin using solid lipid nanoparticles. Food Chem. 2020;302:125328. 31404868 10.1016/j.foodchem.2019.125328

[24] UniProt. The universal protein knowledgebase in 2023. Nucleic Acids Res. 2023;51(D1):D523–31. 36408920 10.1093/nar/gkac1052 PMC9825514

[25] Szklarczyk D, Morris JH, Cook H, Kuhn M, Wyder S, Simonovic M, Santos A, Doncheva NT, Roth A, Bork P, Jensen LJ, von Mering C. The STRING database in 2017: quality-controlled protein-protein association networks, made broadly accessible. Nucleic Acids Res. 2017;45(D1):D362–8. 27924014 10.1093/nar/gkw937 PMC5210637

[26] Shannon P, Markiel A, Ozier O, Baliga NS, Wang JT, Ramage D, Amin N, Schwikowski B, Ideker T. Cytoscape: a software environment for integrated models of biomolecular interaction networks. Genome Res. 2003;13(11):2498–504. 14597658 10.1101/gr.1239303 PMC403769

[27] Huang da W, Sherman BT, Lempicki RA. Systematic and integrative analysis of large gene lists using DAVID bioinformatics resources. Nat Protoc. 2009;4(1):44–57. 19131956 10.1038/nprot.2008.211

[28] Trott O, Olson AJ. AutoDock vina: improving the speed and accuracy of Docking with a new scoring function, efficient optimization, and multithreading. J Comput Chem. 2010;31(2):455–61. 19499576 10.1002/jcc.21334 PMC3041641

[29] Berman HM, Westbrook J, Feng Z, Gilliland G, Bhat TN, Weissig H, Shindyalov IN, Bourne PE. Protein Data Bank Nucleic Acids Res. 2000;28(1):235–42. 10592235 10.1093/nar/28.1.235 PMC102472

[30] Liu ZW, Luo ZH, Meng QQ, Zhong PC, Hu YJ, Shen XL. Network pharmacology-based Investigation on the mechanisms of action of Morinda officinalis how. In the treatment of osteoporosis. Comput Biol Med. 2020;127:104074. 33126122 10.1016/j.compbiomed.2020.104074

[31] Akhtari M, Zargar SJ, Vojdanian M, Ashraf-Ganjouei A, Javinani A, Hamzeh E, Rezaiemanesh A, Jamshidi A, Mahmoudi M. P2 receptors mRNA expression profiles in macrophages from ankylosing spondylitis patients and healthy individuals. Int J Rheum Dis. 2020;23(3):350–7. 31884692 10.1111/1756-185X.13783

[32] Yan L, Li Y, Fan F, Gou M, Xuan F, Feng W, Chithanathan K, Li W, Huang J, Li H, Chen W, Tian B, Wang Z, Tan S, Zharkovsky A, Hong LE, Tan Y, Tian L. CSF1R regulates schizophrenia-related stress response and vascular association of microglia/macrophages. BMC Med. 2023;21(1):286. 37542262 10.1186/s12916-023-02959-8 PMC10403881

[33] Sheng X, Xu J, Sun Y, Zhao J, Cao Y, Jiang L, Wu T, Lu H, Duan C, Hu J. Quantitative biochemical phenotypic heterogeneity of macrophages after Myelin debris phagocytosis at a single cell level by synchrotron radiation fourier transform infrared microspectroscopy. Anal Chim Acta. 2023;1271:341434. 37328242 10.1016/j.aca.2023.341434

[34] Li H, Eyo UB. The diversity, destiny, and memory of dams. Immunity. 2024;57(2):200–2. 38354699 10.1016/j.immuni.2024.01.004

[35] Konstantin Nissen S, Farmen K, Carstensen M, Schulte C, Goldeck D, Brockmann K, Romero-Ramos M. Changes in CD163+, CD11b+, and CCR2 + peripheral monocytes relate to parkinson’s disease and cognition. Brain Behav Immun. 2022;101:182–93. 35026420 10.1016/j.bbi.2022.01.005

[36] Nag S, Manias J, Eubanks JH, Stewart DJ. Increased expression of vascular endothelial growth Factor-D following brain injury. Int J Mol Sci 2019;20(7). 10.3390/ijms20071594 PMC6479775 30935023

[37] Mai CL, Tan Z, Xu YN, Zhang JJ, Huang ZH, Wang D, Zhang H, Gui WS, Zhang J, Lin ZJ, Meng YT, Wei X, Jie YT, Grace PM, Wu LJ, Zhou LJ, Liu XG. CXCL12-mediated monocyte transmigration into brain perivascular space leads to neuroinflammation and memory deficit in neuropathic pain. Theranostics. 2021;11(3):1059–78. 33391521 10.7150/thno.44364 PMC7738876

[38] Jiang MZ, Li C, Mao CM, Yu H, Zhou YC, Pu SQ, Li RZ, Liao YJ, Zhang DY, Yang P, Li MH, Li M. The MAPK/ERK signaling pathway involved in Raddeanin A induces apoptosis via the mitochondrial pathway and G2 phase arrest in multiple myeloma. Sci Rep. 2024;14(1):29061. 39580496 10.1038/s41598-024-76465-z PMC11585587

[39] Ellis A, Bennett DL. Neuroinflammation and the generation of neuropathic pain. Br J Anaesth. 2013;111(1):26–37. 23794642 10.1093/bja/aet128

[40] Shi Y, Zhang X, Fang Q, Zhan H, Wang X, Huang X, Fan T, Liu W, Wu W. LANCL1 as the key immune marker in neuropathic pain. Neural Plast. 2022;2022:9762244. 35510269 10.1155/2022/9762244 PMC9061068

[41] Bouhassira D, Lantéri-Minet M, Attal N, Laurent B, Touboul C. Prevalence of chronic pain with neuropathic characteristics in the general population. Pain. 2008;136(3):380–7. 17888574 10.1016/j.pain.2007.08.013

[42] Baron R. Mechanisms of disease: neuropathic pain–a clinical perspective. Nat Clin Pract Neurol. 2006;2(2):95–106. 16932531 10.1038/ncpneuro0113

[43] Papalexi E, Satija R. Single-cell RNA sequencing to explore immune cell heterogeneity. Nat Rev Immunol. 2018;18(1):35–45. 28787399 10.1038/nri.2017.76

[44] Zhang Q, Yu B, Zhang Y, Tian Y, Yang S, Chen Y, Wu H. Combination of single-cell and bulk RNA seq reveals the immune infiltration landscape and targeted therapeutic drugs in spinal cord injury. Front Immunol. 2023;14:1068359. 36742334 10.3389/fimmu.2023.1068359 PMC9894719

[45] Silvin A, Uderhardt S, Piot C, Da Mesquita S, Yang K, Geirsdottir L, Mulder K, Eyal D, Liu Z, Bridlance C, Thion MS, Zhang XM, Kong WT, Deloger M, Fontes V, Weiner A, Ee R, Dress R, Hang JW, Balachander A, Chakarov S, Malleret B, Dunsmore G, Cexus O, Chen J, Garel S, Dutertre CA, Amit I, Kipnis J, Ginhoux F. Dual ontogeny of disease-associated microglia and disease inflammatory macrophages in aging and neurodegeneration. Immunity. 2022;55(8):1448–e14656. 35931085 10.1016/j.immuni.2022.07.004

[46] Luo S, Wang L, Xiao Y, Cao C, Liu Q, Zhou Y. Single-Cell RNA-Sequencing integration analysis revealed immune cell heterogeneity in five human autoimmune diseases. BIO Integr 2023;4(4).

[47] Li D, Yang K, Li J, Xu X, Gong L, Yue S, Wei H, Yue Z, Wu Y, Yin S. Single-cell sequencing reveals glial cell involvement in development of neuropathic pain via Myelin sheath lesion formation in the spinal cord. J Neuroinflammation. 2024;21(1):213. 39217340 10.1186/s12974-024-03207-3 PMC11365210

[48] Feng R, Muraleedharan Saraswathy V, Mokalled MH, Cavalli V. Self-renewing macrophages in dorsal root ganglia contribute to promote nerve regeneration. Proc Natl Acad Sci U S A. 2023;120(7):e2215906120. 36763532 10.1073/pnas.2215906120 PMC9963351

[49] Liu T, Li T, Chen X, Zhang K, Li M, Yao W, Zhang C, Wan L. A network-based analysis and experimental validation of traditional Chinese medicine Yuanhu Zhitong formula in treating neuropathic pain. J Ethnopharmacol. 2021;274:114037. 33746000 10.1016/j.jep.2021.114037

[50] Zhou MY, Yao CH, Yang YJ, Li X, Yang J, Liu JH, Yu BY, Dai WL. Based on spinal central sensitization creating analgesic screening approach to excavate anti-neuropathic pain ingredients of corydalis Yanhusuo w.t.wang. J Ethnopharmacol. 2024;319Pt 1:117084. 10.1016/j.jep.2023.117084 37666376

[51] Zhang W, Huai Y, Miao Z, Qian A, Wang Y. Systems Pharmacology for investigation of the mechanisms of action of traditional Chinese medicine in drug discovery. Front Pharmacol. 2019;10:743. 31379563 10.3389/fphar.2019.00743 PMC6657703

[52] Tonello R, Silveira Prudente A, Hoon Lee S, Faith Cohen C, Xie W, Paranjpe A, Roh J, Park CK, Chung G, Strong JA, Zhang JM, Berta T. Single-cell analysis of dorsal root ganglia reveals metalloproteinase signaling in satellite glial cells and pain. Brain Behav Immun. 2023;113:401–14. 37557960 10.1016/j.bbi.2023.08.005 PMC10530626

[53] Wang K, Wang S, Chen Y, Wu D, Hu X, Lu Y, Wang L, Bao L, Li C, Zhang X. Single-cell transcriptomic analysis of somatosensory neurons uncovers Temporal development of neuropathic pain. Cell Res. 2021;31(8):904–18. 33692491 10.1038/s41422-021-00479-9 PMC8324866

[54] Soong J, Chen Y, Shustef EM, Scott GA. Sema4D, the ligand for Plexin B1, suppresses c-Met activation and migration and promotes melanocyte survival and growth. J Invest Dermatol. 2012;132(4):1230–8. 22189792 10.1038/jid.2011.414 PMC3305852

[55] Gosselin RD, Meylan P, Decosterd I. Extracellular microvesicles from astrocytes contain functional glutamate transporters: regulation by protein kinase C and cell activation. Front Cell Neurosci. 2013;7:251. 24368897 10.3389/fncel.2013.00251 PMC3857901

[56] Zhao C, Leitges M, Gereau RWt. Isozyme-specific effects of protein kinase C in pain modulation. Anesthesiology. 2011;115(6):1261–70. 22042410 10.1097/ALN.0b013e3182390788 PMC3226912

[57] Matsuoka Y, Yang J. Selective Inhibition of extracellular signal-regulated kinases 1/2 blocks nerve growth factor to brain-derived neurotrophic factor signaling and suppresses the development of and reverses already established pain behavior in rats. Neuroscience. 2012;206:224–36. 22280975 10.1016/j.neuroscience.2012.01.002 PMC3578510

[58] Xu JT, Tu HY, Xin WJ, Liu XG, Zhang GH, Zhai CH. Activation of phosphatidylinositol 3-kinase and protein kinase b/akt in dorsal root ganglia and spinal cord contributes to the neuropathic pain induced by spinal nerve ligation in rats. Exp Neurol. 2007;206(2):269–79. 17628541 10.1016/j.expneurol.2007.05.029

[59] Sun RQ, Tu YJ, Yan JY, Willis WD. Activation of protein kinase b/akt signaling pathway contributes to mechanical hypersensitivity induced by capsaicin. Pain. 2006;120(1–2):86–96. 16360265 10.1016/j.pain.2005.10.017

[60] Chouhan D, Akhilesh V, Tiwari. Focal adhesion kinase Inhibition ameliorates burn Injury-Induced chronic pain in rats. Mol Neurobiol. 2025;62(4):4466–83. 39460902 10.1007/s12035-024-04548-z

[61] Hunger-Glaser I, Salazar EP, Sinnett-Smith J, Rozengurt E. Bombesin, lysophosphatidic acid, and epidermal growth factor rapidly stimulate focal adhesion kinase phosphorylation at Ser-910: requirement for ERK activation. J Biol Chem. 2003;278(25):22631–43. 12692126 10.1074/jbc.M210876200

[62] Gong Z, Zhang Y, Wang W, Li X, Wang K, You X, Wu J. Netrin-1 role in nociceptive neuron sprouting through activation of DCC signaling in a rat model of bone cancer pain. J Integr Neurosci. 2024;23(3):47. 38538215 10.31083/j.jin2303047

[63] Fang Y, Zhang T, Li L, Chen S, Wang L, Tang J, Liao Y. Nicotine Decreases Nerve Regeneration and Pain Behaviors via PTEN and Downstream Inflammation-Related Pathway in Two Rat Nerve Injury Models, eNeuro 10(9) (2023). 10.1523/ENEURO.0185-23.2023 PMC10484360 37620149

[64] Han B, Zhou S, Zhang Y, Chen S, Xi W, Liu C, Zhou X, Yuan M, Yu X, Li L, Wang Y, Ren H, Xie J, Li B, Ju M, Zhou Y, Liu Z, Xiong Z, Shen L, Zhang Y, Bai Y, Chen J, Jiang W, Yao H. Integrating Spatial and single-cell transcriptomics to characterize the molecular and cellular architecture of the ischemic mouse brain. Sci Transl Med. 2024;16(733):eadg1323. 38324639 10.1126/scitranslmed.adg1323

[65] Ponta H, Sherman L, Herrlich PA. CD44: from adhesion molecules to signalling regulators. Nat Rev Mol Cell Biol. 2003;4(1):33–45. 12511867 10.1038/nrm1004

[66] Huang BB, Zhang YJ, Ruan GF, Yu X, Liu Q, Zhang MJ, Yu MZ, Chen A, Liang YB, Xie LD, Luo L. The impact of SGLT1 Inhibition on frailty and sarcopenia: A mediation Mendelian randomization study. J Cachexia Sarcopenia Muscle. 2024;15(6):2693–704. 39474649 10.1002/jcsm.13614 PMC11634476

[67] Wang Y, Yuan Y, Wang W, He Y, Zhong H, Zhou X, Chen Y, Cai XJ, Liu LQ. Mechanisms underlying the therapeutic effects of Qingfeiyin in treating acute lung injury based on GEO datasets, network Pharmacology and molecular Docking. Comput Biol Med. 2022;145:105454. 35367781 10.1016/j.compbiomed.2022.105454

[68] Morris GM, Huey R, Lindstrom W, Sanner MF, Belew RK, Goodsell DS, Olson AJ. AutoDock4 and AutoDockTools4: automated Docking with selective receptor flexibility. J Comput Chem. 2009;30(16):2785–91. 19399780 10.1002/jcc.21256 PMC2760638

[69] Nayak R, Mallick B. BMS345541 is predicted as a repurposed drug for the treatment of TMZ-resistant glioblastoma using target gene expression and virtual drug screening. Cancer Genet. 2024;288–289:20–31. 39213700 10.1016/j.cancergen.2024.08.082

